# New Therapeutic Strategies for Adult Acute Myeloid Leukemia

**DOI:** 10.3390/cancers14112806

**Published:** 2022-06-05

**Authors:** Hiroto Ishii, Shingo Yano

**Affiliations:** Division of Clinical Oncology & Hematology, Department of Internal Medicine, The Jikei University School of Medicine, Tokyo 1058461, Japan; h-ishii@jikei.ac.jp

**Keywords:** acute myeloid leukemia, FLT3 inhibitor, targeted therapy, precision medicine

## Abstract

**Simple Summary:**

For almost 40 years, the combination of anthracyclines and cytarabine, called 3 + 7, has been the standard of induction chemotherapy for the treatment of acute myeloid leukemia (AML). However, with the advent of new drugs in recent years, it has become possible to improve the prognosis of patients with AML harboring certain genetic mutations. Additionally, immunotherapies and therapies targeting cell-surface antigens, which are highly expressed in AML, are emerging. Herein, we review new therapeutic strategies for AML that are evolving with the introduction of these drugs.

**Abstract:**

Acute myeloid leukemia (AML) is a genetically heterogeneous hematological malignancy. Chromosomal and genetic analyses are important for the diagnosis and prognosis of AML. Some patients experience relapse or have refractory disease, despite conventional cytotoxic chemotherapies and allogeneic transplantation, and a variety of new agents and treatment strategies have emerged. After over 20 years during which no new drugs became available for the treatment of AML, the CD33-targeting antibody–drug conjugate gemtuzumab ozogamicin was developed. This is currently used in combination with standard chemotherapy or as a single agent. CPX-351, a liposomal formulation containing daunorubicin and cytarabine, has become one of the standard treatments for secondary AML in the elderly. FMS-like tyrosine kinase 3 (*FLT3*) inhibitors and isocitrate dehydrogenase 1/2 (*IDH* 1/2) inhibitors are mainly used for AML patients with actionable mutations. In addition to hypomethylating agents and venetoclax, a B-cell lymphoma-2 inhibitor is used in frail patients with newly diagnosed AML. Recently, tumor protein p53 inhibitors, cyclin-dependent kinase inhibitors, and NEDD8 E1-activating enzyme inhibitors have been gaining attention, and a suitable strategy for the use of these drugs is required. Antibody drugs targeting cell-surface markers and immunotherapies, such as antibody–drug conjugates and chimeric antigen receptor T-cell therapy, have also been developed for AML.

## 1. Introduction

Acute myeloid leukemia (AML) is a genetically heterogeneous hematologic malignancy characterized by the monoclonal proliferation of myeloid progenitor cells and their impaired differentiation into mature cells. Table 1 shows the European LeukemiaNet (ELN) and the National Comprehensive Cancer Network (NCCN) AML prognostic classifications [1,2], both of which indicate the importance of chromosomal and genetic analysis results in the diagnosis of AML. Although it is assumed that chromosomal abnormalities would be detected in most AML cases [3], about 50% of them are diagnosed as a normal karyotype, even when using high-precision staining such as FISH and SKY methods [4,5,6]. In other words, a chromosome analysis alone is inadequate for the detailed prediction of prognosis, since about 50% of AML patients are lumped into one group. Thus, a genetic analysis is important to stratify the prognosis of patients more finely.

The ELN risk stratification and the NCCN guidelines both classify patients with AML into three groups: favorable, intermediate, and poor/adverse, focusing on chromosomal and genetic abnormalities for which prognostic evaluation has been established. These risk classifications will likely change as newer therapies become available. CBF-AML with t(8;21), inv(16), or t(16;16) is classified as a favorable risk even if other chromosomal and genetic abnormalities are present. However, KIT mutations are associated with a worse prognosis. It has also been reported that the prognosis of patients with triple mutations, such as DNMT3A and NPM1 mutations and FLT3-ITD, is poor, but the prognosis of patients with these less frequent mutations has not been established nor described. Therefore, if mutations other than those listed in Table 1 are found, prognostic evaluation should be performed accordingly.

The Cancer Genome Atlas study [7] found, as in many other neoplasms, multiple driver mutations in individual AML cases. However, the combination of mutations was not random, and there have been many reports of mutations that tend to coexist and those that are present exclusively in AML [8].

In general, multiple genetic abnormalities are associated with poor prognosis, just as complex karyotypes are associated with poor prognosis. AML with the *NPM1* mutation and biallelic mutations in the *CEBPA* gene is associated with a better prognosis than that of AML without mutations. *RUNX1*, *FLT3*-ITD, *TP53*, and *ASXL1* mutations have been adopted as poor prognostic mutations in the ELN risk classification, and should be evaluated at the time of initial diagnosis. Moreover, it has been concluded that *FLT3*-ITD has no prognostic impact in patients with mutant allele ratios of 0.5 or less [1,9,10]. Conversely, the Japan Adult Leukemia Study Group cohort study showed that *FLT3*-ITD AML with low allele ratios also had a poor prognosis, and it is important to note that prognostic impact varied with the chemotherapy regimen used [11]. In addition, the method of *FLT3*-ITD allele measurement has not been standardized in each study, hindering the interpretation of results.

Various genetic abnormalities are known to be involved in AML development. First, initiating mutations in genes related to transcription factors and epigenetic regulation occur in hematopoietic stem cells, resulting in initiating clones. Next, driver mutations in kinase families of the initiating clone expand the clone size, leading to the constant activation of cell proliferation signals and leukemia development. Additionally, multiple passenger mutations can be acquired with the emergence of numerous subclones within the leukemia, which are thought to be involved in drug resistance and relapse. AML has been thought to clinically develop when a pre-leukemic clone emerges with *NPM1*, *DNMT3A*, *IDH*, *RUNX1*, *FLT3*, or *RAS* mutations, which are identified with a frequency of more than 10%, and when additional founder mutations occur [12]. Therefore, the development of therapeutic agents targeting these clones is expected to be a potential treatment strategy for AML.

Precision medicine has enabled the analysis of tumor cell genomic information and the selection of treatments based on its results. However, the rapid initiation of treatment is often required for AML, hindering the introduction of genomic medicine based on genetic analysis due to its time-consuming nature.

The German-Austrian AML Study Group (AMLSG) carried out a prospective study to stratify treatments by analyzing mutations in *RUNX1*-*RUNX1T1*, *CBFB-MYH11*, *PML-RARA*, *KIT*, *FLT3*-ITD, *NPM1*, and *MLL* within 48 h of initial diagnosis. After stratification, the results were incorporated into clinical trials, including all-trans retinoic acid (ATRA) plus arsenic trioxide for acute promyelocytic leukemia (APL0406, [13]), and *FLT3*-ITD-positive AML treated with midostaurin plus chemotherapy and allogeneic transplantation (AMLSG16-10, [14]), among several others.

The Leukemia and Lymphoma Society of the United States reported the results of the Beat AML study, which evaluated the feasibility of cytogenetic testing and genetic analysis within seven days of enrollment and treatment based on their results in patients with AML aged 60 years and older [15]. Using bone marrow samples, cytogenetic analysis, *FLT3*-ITD testing, and targeted sequencing using the FoundationOne Heme platform were performed. The Beat AML study assigned 11 substudies based on the results of genetic analysis. From November 2016 to January 2019, 487 patients were enrolled, and 395 patients met the eligibility criteria, with a median age of 72 years (range 60–92 years). Within seven days, cytogenetic testing and genetic analysis were achieved for 374 patients, who were then assigned to a substudy. In total, 224 patients agreed to the trial treatment. Among the remaining 171 patients, 103 patients received standard treatment, 28 patients chose other investigational treatments, and 38 patients opted for palliative treatment. The latter group tended to have a higher white blood cell count at the time of initial diagnosis than that of other groups, but there were no other differences in patient characteristics. The mortality rate within 30 days was 14.1% (95% confidence interval (CI) 10.9–18.1%) for the entire cohort, and 7.5% (95% CI 5.1–10.9%) excluding patients in palliative care.

The 30-day mortality rate was 3.7% (95% CI 1.9–7.2%) for the 224 patients who chose to enroll in the Beat AML trial, and 20.4% (95% CI 13.0–31.2%) for the 103 patients on standard treatment. The overall survival (OS) in the Beat AML trial treatment group was 12.8 months, significantly longer than that of the standard treatment group (3.9 months) and that of the palliative treatment group (0.6 months), suggesting the feasibility of precision medicine for AML.

**Figure 1:**
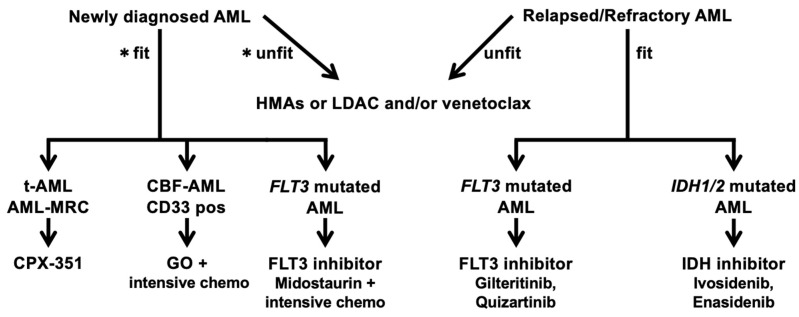
Treatment algorithm of AML. AML, acute myeloid leukemia; HMAs, hypomethylating agents; LDAC, low-dose cytarabine; t-AML, therapy-related AML; AML-MRC, AML with myelodysplasia-related changes; CBF-AML, core-binding factor AML. * Conceptual criteria for selecting unfit patients. 1. advanced age; 2. organ dysfunction and comorbidity(heart, lung, kidney, liver); 3. active infection; 4. congnitive impairment; 5. low performance status; 6. socio-economical issue.

The median observation period in the Beat AML study was short (7.1 months), and the breakdown of standard treatment was unclear. The OS after treatment with the combination of azacitidine and venetoclax for AML in the elderly and unfit, which will be described separately in this review, was 14.7 months [16]. Although flexibility in response to patient age and genetic analysis results is required, these results are important in considering new treatment strategies for AML. With the advent of new drugs, AML patients who have been refractory to standard chemotherapy can now be expected to have an improved prognosis. However, the types of genetic mutations that can be searched for, the accuracy of the tests, and the approval status of new drugs vary across countries and institutions. The purpose of this review is to provide information that will be useful when these new agents become available. Although the evaluation of each drug will change with accumulating evidence, we present expert views on therapeutic strategies to be used in this era of new agents, based on data reported to date (Table 2 and Figure 1).

The treatment algorithm (Table 1) begins by assessing whether the patient is fit or unfit for the standardof treatment. Then, targeted therapy is recommended while checking the availability of drugs in the relevant country. This figure will be rewritten in the future when TP53 inhibitors and CDKinhibitors have the same level of evidence as other targeted therapies.

The human immune system is responsible for suppressing disordered cell growth in the body and inhibiting the progression of cancer. However, in patients with cancer, the cells and molecules of the immune system that are responsible for preventing cancer growth are often severely compromised. Various studies and drug discovery efforts have been carried out in an attempt to elicit a functional immune response against cancer [17]. In hematological malignancies, as in solid tumors, the clinical application of immunotherapy is being attempted. Anti-PD-1 antibody therapy for Hodgkin’s lymphoma [18,19], anti-CD19 chimeric antigen receptor (CAR)-T cell therapy [20,21,22] and bispecific antibody therapy for B-cell tumors [23,24] have already been approved by the FDA for clinical use. The development of immunotherapy for AML is also underway and will be introduced at the end of this review.

## 2. Cytotoxic Agents for AML

### CPX-351

CPX-351 is a liposomal formulation of daunorubicin and cytarabine, which are structurally similar to cell membranes and can be efficiently taken up by cells and released into leukemic cells, leading to cell death. Thanks to liposomalization, CPX-351 has a longer half-life in the blood and accumulates in the bone marrow, enabling it to be selectively delivered to leukemia cells, thus, exerting a high anti-leukemic effect [25,26].

A phase 3, randomized, controlled clinical trial of CPX-351 vs. conventional remission-induction therapy in untreated patients with secondary AML aged 60–75 years was conducted [27]. Secondary AML included therapy-related AML, AML evolving from myelodysplastic syndrome (MDS) and chronic myelomonocytic leukemia (CMML), and AML with MDS-related chromosomal aberrations. CPX-351 was administered at 100 units/m^2^/day on Days 1, 3, and 5 as remission-induction therapy. Conventional induction therapy included daunorubicin 60 mg/m^2^/day administered on Days 1–3 and cytarabine 100 mg/m^2^/day administered on Days 1–7. The primary endpoint was OS, with a median of 9.56 vs. 5.95 months in the CPX-351 vs. conventional remission-induction therapy groups, respectively (hazard ratio (HR) 0.69; 95% CI 0.52–0.90, *p* = 0.003), confirming the prolonging effect. The CPX-351 group had equally favorable outcomes in remission rate (47.7% vs. 33.3%, *p* = 0.016) and event-free survival (median 2.53 vs. 1.31 months, HR = 0.74, *p* = 0.021). In a subgroup analysis, CPX-351 showed improved survival in patients with treatment-related AML and AML that progressed from MDS or CMML, patients with favorable or intermediate chromosomal prognosis, and patients with negative *FLT3* mutations. However, patients on CPX-351 required more time to recover blood cell counts than patients on conventional remission-induction therapy (median time to reach 500 neutrophils/µL, 35 vs. 29 days, respectively; median time to reach 50,000 platelets/µL, 36.5 vs. 29 days, respectively). The frequency of adverse events was similar between the CPX-351 and 3 + 7 groups, but the CPX-351 group reported a longer duration of adverse events due to the longer duration of treatment. After adjusting for these factors, the frequency and duration of adverse events in the CPX-351 and 3 + 7 groups were similar. The most common adverse events were febrile neutropenia (68.0% vs. 70.9%), pneumonia (19.6% vs. 14.6%), and hypoxia (13.1% vs. 15.2%). Early mortality rates with CPX-351 and 3 + 7 groups were 5.9% and 10.6% (two-sided *p* = 0.149) until Day 30 and 13.7% and 21.2% (two-sided *p* = 0.097) until Day 60, respectively [27].

Based on these results, the NCCN guidelines strongly recommend CPX-351 as the first-line treatment for secondary AML in the elderly [2]. The proportion of secondary AML among elderly AML cases is higher than that among younger cases, and CPX-351 is an effective drug for elderly patients with good organ function, activities of daily living (ADL), and cognitive function. However, even though its OS benefit is significantly different from that of existing cell-killing drugs, CPX-351 only extends the OS by 4 months. Therefore, a strategy using appropriate targeted therapy as ground-up or maintenance therapy with genetic mutation analysis, whenever possible, should be considered.

## 3. Genetic-Targeted Strategies for AML

### 3.1. FLT3 Inhibitors

FMS-like tyrosine kinase 3 (*FLT3*) is a receptor-type tyrosine kinase expressed on the cell surface of hematopoietic cells, and is involved in cell differentiation and proliferation. Internal tandem duplication (ITD), in which a portion of the transmembrane region of the *FLT3* gene is duplicated and repeated, and a point mutation in the tyrosine kinase domain (TKD mutation) of the *FLT3* gene cause ligand-independent homeostatic activation of the FLT3 receptor. This results in abnormal cell proliferation and the inhibition of differentiation, and contributes to the development of AML. *FLT3*-ITD and *FLT3*-TKD mutations are found in about 30% and 10% of AML cases, respectively [28,29]. *FLT3*-ITD is a poor prognostic factor for AML. Among 224 patients enrolled in two multicenter clinical trials, grouped by the presence or absence of *FLT3*-ITD, patients with *FLT3*-ITD AML had significantly shorter remission durations and shorter OS than those without *FLT3*-ITD [30].

*FLT3*-ITD is also classified as a poor prognostic genetic variant in the ELN and NCCN guidelines [1,2]. *FLT3*-ITD has a different prognostic significance depending on its coexisting gene mutations. The presence of *NPM1* mutations in patients with a high *FLT3*-ITD allele ratio (the ratio of *FLT3*-ITD to wild-type *FLT3*) is considered to be related to an intermediate prognosis. Patients with a low *FLT3*-ITD allele ratio are placed in the favorable prognosis group [1].

*FLT3*-TKD mutations have been evaluated as a prognostic factor in AML; some reports have shown that *FLT3*-TKD mutations are associated with shorter remission and OS, while others have shown that *FLT3*-TKD mutations do not affect prognosis or display a favorable prognosis [31,32,33]. These differences in results may be due to differences in patient backgrounds and treatment methods.

FLT3 inhibitors are classified based on their specificity for and mechanism of interaction with FLT3. First-generation inhibitors have low specificity for FLT3 and inhibit many other receptor tyrosine kinases as an off-target, and second-generation inhibitors have high specificity for *FLT3* with few off-targets. Furthermore, type 1 FLT3 inhibitors are effective against both *FLT3*-ITD and *FLT3*-TKD mutations. Type 2 FLT3 inhibitors are effective only for *FLT3*-ITD and not *FLT3*-TKD mutations, as they cause FLT3 receptors to be permanently activated.

Midostaurin is a first-generation type 1 inhibitor that inhibits tyrosine kinases such as VEGFR2, PDGFRβ, and RET as off-targets. The RATIFY trial was a multicenter, randomized, placebo-controlled, double-blind study evaluating the effect of midostaurin in combination with standard chemotherapy in patients with newly diagnosed *FLT3*-mutated AML [34]. Patients with *FLT3* mutation-positive AML were randomized to receive midostaurin (360 patients) or a placebo (357 patients). Patients were randomized according to high allele ratio, low allele ratio, and *FLT3*-TKD mutation positivity in *FLT3*-ITD mutation-positive patients. The primary endpoint was OS and patients received remission induction with daunorubicin plus cytarabine and consolidation with high-dose cytarabine. In the midostaurin group, 50 mg of midostaurin was administered twice daily. The remission rate was 58.9% in the midostaurin group and 53.5% in the placebo group, with no significant differences between the two groups (*p* = 0.15). However, both remission rates were lower than those of the historical cohorts, because the definition of remission was different from that of other trials. This point should be interpreted with caution. The median OS was 74.7 months in the midostaurin group and 25.6 months in the placebo group (HR 0.78, 95% CI 0.63–0.96, *p* = 0.009). The 4-year OS was 51.4% in the midostaurin group and 44.3% in the placebo group. For patients who underwent allogeneic transplantation, the OS rate was 63.7% and 55.7% in the midostaurin and placebo groups, respectively, with midostaurin showing a nonsignificant improvement (*p* = 0.08). Additionally, a post hoc subset analysis of 174 patients who subsequently underwent maintenance therapy, with a landmark analysis at the start of maintenance therapy, showed no significant differences in event-free survival between the midostaurin and placebo groups during or after maintenance therapy [35].

Gilteritinib is a second-generation type 1 inhibitor that inhibits tyrosine kinases such as LTK, ALK, and AXL as off-targets. A collaborative, open-label, randomized trial was conducted at 107 sites in 14 countries (ADMIRAL trial [36]). In a phase I/II clinical trial [37], gilteritinib doses (80 mg/day or higher and 40 mg/day or lower) were compared, and the group receiving 80 mg/day or higher had significantly longer survival. The recommended daily dose is 120 mg, and although up to 200 mg can be administered, it is desirable to maintain the 80 mg dose even when toxicity forces a dose reduction.

In this study, 371 patients with relapsed or refractory *FLT3*-ITD or TKD mutation-positive AML were randomized 2:1 to receive 120 mg/day of gilterinitib (247 patients) or salvage chemotherapy (124 patients). OS and the rate of complete remission (CR) and CR with partial hematologic recovery (CRh) were the primary endpoints. Among all patients, 88.4% of patients were positive for *FLT3*-ITD, 8.4% of patients were positive for the *FLT3*-TKD mutation, and 1.9% of patients showed both mutations. The interim analysis reported 142 patients in the gilteritinib arm who were eligible for response analysis, with a CR/CRh rate of 28.2%, a median time to first CR/CRh of 113.5 days, and a median duration of response of 148.0 days. The median OS reported for the gilteritinib group (9.3 months, HR 0.637, *p* = 0.0007) was significantly longer than that of the salvage chemotherapy group (5.6 months). The 1-year OS was 37.1% and 16.7% and CR/CRh was 34.0% and 15.3% in the gilteritinib and salvage chemotherapy groups, respectively. Electrocardiogram QTc prolongation was observed in 6% of patients.

Quizartinib is a second-generation type 2 inhibitor that inhibits tyrosine kinases such as KIT, PDGFRβ, and RET as off-targets. Quizartinib was approved for the treatment of relapsed or refractory *FLT3*-ITD-positive AML in Japan in 2019. It was administered at doses ranging from 12 to 450 mg/day in a phase 1 study of 76 patients with relapsed or refractory AML, who were included with or without *FLT3*-ITD mutations. The dose-limiting toxicity was grade 3 QTc prolongation, and the maximum tolerated dose was 200 mg/day [38]. However, several patients with QTc prolongation were observed even at 200 mg in a subsequent phase 2 trial [39]. After a phase 2b trial comparing 60 mg and 90 mg quizartinib [40], a phase 3 trial (the QuANTUM-R trial) was performed. Patients were randomized 2:1—245 were assigned to the quizartinib arm and 122 were assigned to the salvage chemotherapy arm [41]. Patients received quizartinib at 30 mg/day for 15 days, titrated to 60 mg in the absence of QTc prolongation. The standard salvage chemotherapy arm consisted of low-dose cytarabine, MEC, and FLAG-IDA, with OS as the primary endpoint. After 23.5 months of observation, the OS were 6.2 and 4.7 months in the quizartinib and chemotherapy groups, respectively (*p* = 0.02). One-year OS was 27% and 20% in the quizartinib and standard chemotherapy groups, respectively. Febrile neutropenia (33%), sepsis (21%), QTc prolongation (26%), and pneumonia (16%) were observed in the quizartinib group. The CR/CRh rate was significantly higher in the quizartinib group (48%) as compared with that in the chemotherapy group (27%) (*p* < 0.0001). The median time to first CR/CRh in the quizartinib group was 4.9 weeks, and the median duration of CR/CRh was 12.1 weeks, suggesting that the patients in the quizartinib group may reach CR/CRh earlier than those in the gilteritinib group. In this study, 32% of patients in the quizartinib group received an allogeneic HSCT after quizartinib treatment, and 62% of patients with allogeneic transplantation received quizartinib after transplantation.

Favorable results are expected from the study of quizartinib in combination with conventional chemotherapy in patients with untreated *FLT3*-ITD mutation-positive AML (NCT02668653).

**Figure 2:**
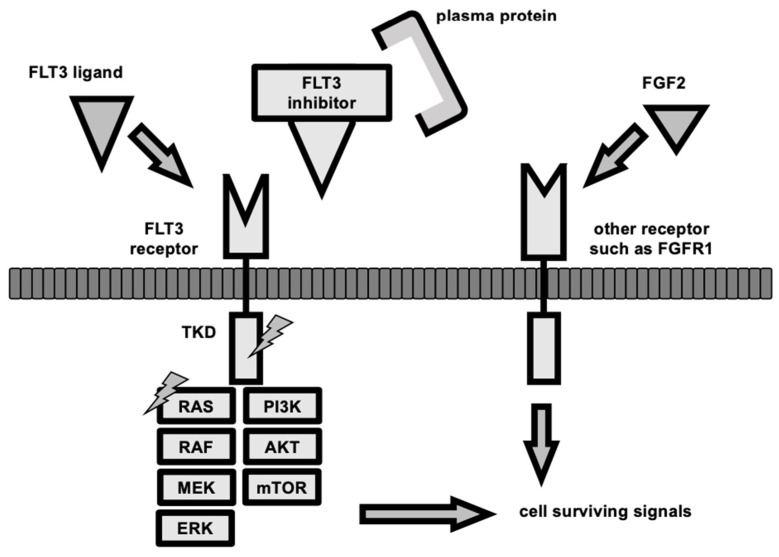
The mechanism of resistance to FLT3 inhibitors. The FLT3 signaling pathway is activated by dimerization and auto-phosphorylation of the FLT3 receptor, which subsequently activates TKD, then PI3/AKT/mTOR signaling, JAK/STAT signaling, and RAS/MAPK signaling to promote cell survival, proliferation, and differentiation. FLT3 inhibitors bind to the FLT3 receptor and block this signaling pathway; however, FLT3 ligand competes with the receptor binding, and fibroblast growth factor 2 (FGF2) promotes signaling from another receptor via fibroblast growth factor receptor 1 (FGFR1). Clones other than AML that proliferate in an FLT3-dependent manner may arise as a result of selective pressure by FLT3 inhibitors or clonal evolution during FLT3 inhibitor treatment. TKD mutations (lightning sign) or RAS/MAPK signaling-related genes mutations (lightnign sign) allow cells to survive without upstream signaling, and thus, become drug-resistant. Some acquired TKD mutations also inhibit the binding of FLT3 inhibitors to their receptors. The binding of plasma proteins, such as Acid-glycoprotein, to FLT3 inhibitors is also known to attenuate the effect of the inhibitors.

Although FLT3 inhibitors are effective against *FLT3*-mutated AML, the duration of the response is not satisfactory. The mechanism of this resistance is known (Figure 2). After chemotherapy, FLT3-ligand (FL) and fibroblast growth factor 2 are overproduced from the stroma and activate FLT3 and FGF1 receptors expressed on leukemic cells, attenuating the effect of FLT3 inhibitors [42,43,44]. The acquisition of a D835 mutation in the FLT3-TKD region is a major mechanism in treatment resistance to type 2 FLT3 inhibitors [45]. There have been reports of genetic mutations occurring in *FLT3*, such as *FLT3*-F691L, a secondary *FLT3*-ITD mutation of the gate-keeper residue F691 [46,47,48]. Multiple TKD mutations (such as Y693C/N and G697S) result in the hampering of FLT3 inhibitor binding to the FLT3 receptor [49]. When selection pressure is applied to FLT3 inhibitors, clones with mutations are selected out, but *FLT3* wild-type AML subclones with other genetic mutations present before administration increase or acquire new non-*FLT3* mutations and develop resistance [50,51]. Genetic mutations associated with the *RAS*/MAPK signaling pathway downstream of *FLT3* emerge, leading to constant *RAS*/MAPK activation in a *FLT3* signaling-independent manner and FLT3 inhibitor resistance [52]. Some plasma proteins bind to FLT3 inhibitors and reduce their efficacy [44].

It should be noted that *FLT3* mutation is a cooperating mutation rather than a driver or initial mutation in AML. All FLT3 inhibitors have a short CR duration, and prolonged single-agent use may lead to the expansion of resistant clones. Therefore, their use is only recommended for a short period of time and as a bridge therapy until the next treatment, such as allogeneic transplantation. Although the results of clinical trials and real-world clinical experience should be taken into consideration, the combination of FLT3 inhibitors, RAS inhibitors, and hypomethylating agents from the early phase of treatment may be an option when considering FLT3 inhibitor resistance mechanisms.

In the open-label phase I/II trial, patients of any age receiving first-salvage treatment for FLT3-ITD AML or age > 60 years with untreated myelodysplastic syndrome or AML were treated with quizartinib plus azacitidine or low-dose cytarabine (LDAC) [53]. Among previously untreated patients, composite response (CRc; CR, CR with incomplete hematologic recovery, and CR without platelet recovery) was achieved in 13/15 (87%) of patients treated with quizartinib/AZA and 14/19 (74%) of patients treated with quizartinib/LDAC. Among previously treated patients, 16/25 (64%) of patients achieved CRc in quizartinib/AZA and 4/14 (29%) of patients achieved CRc in quizartinib/LDAC. These response rates were higher than expected, confirming the preclinical observed synergy. The median OS for untreated and first-salvage treatment was 19.2 months and 12.8 months, respectively. QTc prolongation grade 3 occurred in only one patient in each cohort. Quizartinib combined with azacitidine appears to be effective as both a frontline and first-salvage treatment for FLT3-ITD-mutated patients, and is well-tolerated.

FLT3 inhibitors are also recommended as maintenance therapy after transplantation [54]. Since graft-versus-host disease may worsen after FLT3 inhibitor administration, we ought to wait for clinical trial results and refer to them for the optimal starting time and dose.

### 3.2. IDH 1/2 Inhibitors

Among isocitrate dehydrogenase (*IDH*) genes, *IDH1* and *IDH2* both convert isocitrate to α-ketoglutarate (αKG). *IDH1* is mainly localized in the cytoplasm, whereas *IDH2* is localized in the mitochondria. Mutant *IDH* converts αKG to an abnormal metabolite, hydroxyglutarate, which competitively inhibits αKG binding to several histone demethylase enzymes, including KDM2a. This leads to abnormal histone modification, particularly histone tail methylation. The resulting DNA hypermethylation and increased inhibitory histone methylation may impair the differentiation and maturation of HSCs and promote leukemogenesis. *IDH1* and *IDH2* mutations are found in 6–10% and 15–20% of AML cases, respectively [55,56,57,58]. The prognostic impact of *IDH* mutations has not been established. Inhibitors of mutant *IDH* are drugs that exert antitumor effects by inhibiting the production of hydroxyglutaric acid, thereby, reducing the inhibitory effect on αKG-dependent enzymes and restoring the normal mechanism of action.

A phase 1 trial of ivosidenib, an *IDH1* inhibitor, was conducted on 258 patients with *IDH1* mutation-positive relapsed/refractory AML [59]. In the primary efficacy population (125 patients), the overall response rate was 41.6%, and the complete remission rate was 21.6%. The OS of the group that achieved complete response was 9.3 months. Among patients who achieved CR + CRh, 21% had a deep response with undetectable *IDH* mutations. In a study of 34 newly diagnosed AML patients who received ivosidenib 500 mg daily, the median OS was 12.6 months. Toxicity was similar to that in the clinical study of relapsed/refractory AML, with diarrhea (53%), fatigue (47%) nausea and vomiting (38%) being the most common symptoms, and differentiation syndrome was present in 18% but not severe enough to require treatment discontinuation [60].

A phase 1/2 study of enasidenib, an *IDH2* inhibitor, was conducted involving 176 patients with *IDH2* mutation-positive relapsed/refractory AML [61]. The overall response and complete remission rates were 40.3% and 19.3%, respectively. The OS of the group with complete response was 19.7 months. A clinical trial of 214 patients treated with 100 mg enasidenib after this study reported that 11.9% of patients had a deep molecular response [62].

Due to the mechanism of action of *IDH* inhibitors, differentiation syndrome (DS) is observed in 10–20% of responders. The clinical manifestations are similar to those of ATRA treatment for acute promyelocytic leukemia. However, the onset of ATRA-DS is bimodal, occurring within the first week of treatment and between Days 15 and 28 [63], whereas *IDH* inhibitor-DS may occur as late as 3 to 4 months after treatment initiation. The previously reported median onset was 14.5–30 days (range 1–129 days) after initiation of treatment [64,65,66]. A combination study with chemotherapy for untreated AML (the HOVON150AML study NCT03839771) is currently underway, and results are awaited.

### 3.3. TP53 Inhibitors

A mutation in *TP53*, a tumor-suppressor gene located on the short arm of chromosome 17 (17p13), is a frequently observed abnormality in secondary AML and therapy-related leukemia (about 15–20%) [67]. It is more frequently detected in older cases, and leukemia cases with *TP53* mutations show resistance to chemotherapy and poor prognosis [68,69,70].

In a study examining the relationship between genetic mutations in tumor cells and response to decitabine treatment in 116 adult patients with MDS or AML, 53 patients displayed a decrease in blasts to less than 5% [71]. Surprisingly, all 21 patients with *TP53* mutations were included in the response group, indicating that decitabine can eliminate *TP53* mutation-positive clones. In this study, decitabine was administered in monthly cycles at 20 mg/m^2^/day for 10 consecutive days. Based on this finding, prolonging the administration of decitabine may be effective for *TP53*-mutated AML. However, in another study, there were no differences between 5-day and 10-day decitabine treatment in newly diagnosed elderly AML [72].

The drug APR-246 (eprenetapopt) was developed to reactivate mutant *TP53*. Fifty-two patients with MDS and AML participated in a phase 2 study that assessed the efficacy and safety of eprenetapopt in combination with AZA in patients with high-risk *TP53*-mutated MDS and AML [73]. A total of 18 patients with AML were enrolled, including 11 patients with a blast ratio of 30% or less. In total, 62% of patients with MDS had an overall response, including 47% of patients with complete remission, with a median response duration of 10.4 months. Furthermore, 33% of patients with AML had an overall response, including 17% of patients showing complete remission. Among patients with a response, 73% of patients had a molecular response in which the *TP53* mutation could not be identified by next-generation sequencing. The major adverse events were febrile neutropenia and neurological adverse events. After a median observation period of 9.7 months, the median survival was 12.1 months, 13.9 months, and 3.0 months in MDS, AML (with a blast ratio of less than 30%), AML (with blast ratio of 30% or more), respectively. The combination of eprenetapopt and AZA was considered effective in TP53-mutated MDS and AML, especially in patients with a low blast percentage [73].

The clone size of *TP53* mutations in AML is almost always larger than the clone size of concomitant mutations or copy number variants, leading to the emergence of driver *TP53* mutations and subsequent genomic instability. Therefore, based on the results of the abovementioned studies, the use of epenetapopt may be considered in AML with a high tumor burden after reducing the blast percentage or eliminating other clones by means of other treatments such as cytotoxic agents and allogeneic transplantation.

## 4. Non-Genetic-Targeted Strategies for AML

### 4.1. Bcl2 Inhibitors

*BCL2* family members, including Bcl-2, Bcl-XL, and Mcl-1, promote cell survival by binding to and sequestering proteins that promote apoptosis in tumor cells. Bcl-2 is a key regulator of the mitochondrial apoptotic pathway, and plays an important role in the survival and maintenance of AML cells [74,75,76,77]. Consequently, Bcl-2 has been shown to be involved in resistance to chemotherapy and to enhance the survival of leukemic blasts and progenitor cells [78,79]. Bcl-2 sequesters BAX, which promotes apoptosis, thereby maintaining the survival of myeloblasts. When Bcl-2 is inhibited, BAX is released, triggering increased permeability of the mitochondrial outer membrane and cell death.

Venetoclax, an oral medication, is a potent and selective Bcl-2 inhibitor used for multiple myeloma and chronic lymphocytic leukemia [80,81,82,83]. Venetoclax monotherapy in patients with relapsed/refractory AML had an overall response rate of 19% and was well-tolerated [84], despite the suggestion of the involvement of *MCL-1* expression in resistance to this treatment [85,86]. Preclinical studies suggest that various drugs, including anthracyclines, hypomethylating agents (HMAs), and cytarabine, negatively regulate *MCL-1* expression. The combination of these agents with venetoclax is expected to lead to therapeutic synergy [87,88,89,90,91].

The Viale-A study, an international phase 3, randomized, placebo-controlled, double-blind, comparative clinical trial, evaluated the efficacy of venetoclax and azacitidine combination therapy [16] and placebo and azacitidine in patients with newly diagnosed AML who were not eligible for intensive remission-induction therapy. Patients were randomized to receive either venetoclax or placebo orally once daily for 28 days, and azacytidine subcutaneously or intravenously once daily for 7 days. Patients between the ages of 18 and 74 years were deemed ineligible for standard induction therapy with anthracyclines and cytarabine, and patients aged 75 years or older with an Eastern Cooperative Oncology Group Performance Status (ECOG-PS) less than or equal to 2 and normal liver and kidney function regardless of age, were enrolled. The primary endpoints were the composite complete remission rate (i.e., CR and CR with incomplete hematologic recovery (CRi)) and OS. The median age of both arms was 76 years, and ECOG-PS scores of 0 and 1 were found in 55% and 56% of patients in the venetoclax and placebo arms, respectively. The proportions of patients who had received red blood cell and platelet transfusions prior to therapy initiation were 54.2% and 55.9% in the venetoclax and placebo arms, respectively. The median OS was 14.7 months in the venetoclax group and 9.6 months in the placebo group, demonstrating the superiority of the venetoclax/azacitidine combination. CR and CRi rates were significantly higher in the venetoclax group (65.3% vs. 25.3% in the placebo group). CR and CRi were maintained before the start of the second cycle by 7.6% of patients in the placebo group and 43.4% of those in the venetoclax group (*p* < 0.001). The median time to first remission was 1.3 months and 2.8 months, and the duration of CR and CRi was 17.5 months and 13.4 in the venetoclax and placebo groups, respectively. The transfusion-independent rate of red blood cells and platelets in the venetoclax group (58.0%) was significantly higher than in the placebo group (33.8%), and more patients in the venetoclax group did not require transfusion therapy over 8 weeks. Because venetoclax is expected to have an early antitumor effect, prophylaxis should be administered to prevent the development of tumor lysis syndrome. The combination of azacitidine and venetoclax may be useful based on this study, in contrast with a separate phase 3 trial of azacitidine which showed a CR and CRi of 27.8% and a median OS of 10.4 months [92].

The Viale-C trial, an international phase 3, randomized, placebo-controlled, double-blind clinical trial, examined the efficacy of venetoclax in combination with low-dose cytarabine [93]. As in the Viale-A trial, it involved patients with AML not eligible for intensive chemotherapy. The treatment schedule consisted of a 28-day cycle of oral venetoclax or placebo once daily, followed by subcutaneous low-dose cytarabine once daily for 10 days. The primary endpoint was OS. The proportions of patients who had received red blood cell and platelet transfusions before the start of treatment were 74.4% and 36.5%, for the venetoclax and placebo groups, respectively. The median OS was 4.1 months in the placebo group vs. 7.2 months in the venetoclax group, although this difference was not statistically significant. However, a post hoc analysis that extended the follow-up period by 6 months showed a significant prolongation of 8.4 months in venetoclax group vs. 4.1 months in the placebo group. The CR and CRi rates were significantly higher in the venetoclax group as compared with the placebo group (48% vs. 13%).

Venetoclax has been shown to be effective against AML with *IDH1/IDH2* or *RUNX1* mutations in vitro. This was confirmed in the Viale-A study, and in another retrospective study which showed that *IDH* and *RUNX1* mutations were predictors of treatment response after combination therapy with venetoclax and AZA [16,94,95]. Although VEN is considered to be an alternative to conventional cytotoxic chemotherapy for unfit AML patients, more cases must be examined to determine whether VEN is a viable alternative to the so-called ‘3 + 7′ in fit AML patients.

### 4.2. NAE Inhibitors

NEDD8 is the most homologous protein to ubiquitin among the ubiquitin-like proteins (a group of proteins with high structural homology to ubiquitin), reported in 1993 as a group of genes whose expression is repressed during neuroblast differentiation. The major substrate of neddylation is E3 ubiquitin ligase, which is composed of Skp1, Cul1, F-box proteins, and Roc1 complex (SCF ubiquitin ligase complex). The regulation of SCF ubiquitin ligase complex activity by the NEDD8 reaction plays important roles in the regulation of cellular proliferation and differentiation [96,97].

Cullin-dependent activation of RING E3 ubiquitin ligase (CRL) is mediated by NEDD8 activation, which regulates protein metabolism by inducing the timely ubiquitination and proteasomal degradation of proteins that play important roles in cell cycle progression and signaling in tumor cells. NEDD8-activating enzyme (NAE), an E1 enzyme, catalyzes the reaction that binds ATP and NEDD8 to form the NEDD8-AMP intermediate, which is essential for NEDD8 activation. Inhibition of NAE disrupts CRL-mediated protein metabolism and cell cycle S phase regulation, and induces cell death. Preclinical studies have shown that NAE inhibitors are potentially useful in cancer therapy by interfering with the degradation of various important proteins [98,99,100,101].

A single-agent phase 1 study on pevonedistat, an NAE inhibitor, evaluated patients with relapsed/refractory AML or MDS [102,103]. Two dosing schedules, Days 1, 3, and 5 (schedule A) and Days 1, 4, 8, and 11 (schedule B), were both tested with volume titration from 25 mg/m^2^ to 147 mg/m^2^ to find the maximum tolerated dose in one 21-day cycle. In total, 53 patients aged 19 to 84 years were enrolled. The median number of cycles was two, with a maximum tolerated dose of 83 mg/m^2^. Two of the twenty-three patients who received submaximal doses achieved complete remission, and two achieved partial remissions, with an overall response rate of 17%.

A phase 1b trial on pevonedistat in combination with azacitidine evaluated patients with AML not eligible for potent chemotherapy [98]. Sixty-four newly diagnosed patients with AML, aged 61 to 89 years, were enrolled. Pevonedistat was administered on Days 1, 3, and 5 at 20 mg/m^2^, while azacitidine was administered at 75 mg/m^2^ on Days 1–5, 8, and 9 (so-called 5 + 2) in a 28-day cycle. The most common adverse events were constipation (48%), nausea (42%), general malaise (42%), and anemia (39%). Grade 3 or higher adverse events included anemia (30%), febrile neutropenia (30%), and thrombocytopenia (23%). The median response duration was 8.3 months. Of the 32 patients who responded, 20 patients responded in the first two cycles, 14 patients responded after four or more cycles, and 2 received allogeneic HSCT. Pevonedistat has also been shown to induce an upregulation of Noxa [104], given that Noxa has inhibitory effects on MCL-1 [105]. A multicenter phase 1b clinical trial of pevonedistat, azacitidine, and venetoclax (PAVE) therapy is currently underway [106]. Furthermore, alvocidib (flavopiridol), which inhibits cyclin-dependent kinase 9 (CDK9), suppresses MCL-1 by inhibiting CDK9, providing anti-apoptotic and antitumor effects [107]. In an ongoing phase 2 clinical trial on relapsed refractory AML (Zella 201 study [108]), 25 patients with MCL-1 dependence were treated with alvocidib for 3 days, followed by combination chemotherapy with cytarabine and mitoxantrone. The CR/CRi rate was 62%, with CR in 7 of 11 patients with refractory AML. Grade 3 or higher non-hematological toxicities included tumor lysis syndrome (20%) and diarrhea (24%).

### 4.3. CDK Inhibitors

The cell cycle is divided into four periods: G1, S, G2, and M. In the G0 period, cell proliferation is maintained, but cell division is stopped. The complex of cyclin and CDK plays an important role in this progression. There are three types of CDKs: cyclin D1, cyclin D2, and cyclin D3, which are encoded by the *CCND1*, *CCND2*, and *CCND3* genes, respectively. Cyclin D contains a cyclin box domain that mediates CDK binding, an Rb-binding domain at the N-terminal end, and a PEST domain at the C-terminal end, involved in protein stability [109,110,111].

The cyclin D-CDK4/6 complex phosphorylates Rb, releasing E2F, which was previously inactivated by binding to Rb, to become transcriptionally active, promoting transcription of genes required for the transition to the S phase. This process results in cell progression to the S phase. The cyclin D-CDK4/6 complex is negatively regulated by p16INK4A, which is encoded by a respective gene [112].

Tumor cell proliferation is caused by cyclin D mutation and overexpression, CDK4/6 overexpression, and p16INK4A mutation and underexpression, all of which are mediated by the activity of CDK4/6. Therefore, CDK4/6 inhibitors suppress cell proliferation by blocking progression from the G1 to S phase of the cell cycle. Gong et al. investigated the relationship between abemaciclib sensitivity and genetic abnormalities in 560 cell lines derived from various cancers and found that about 15% of the cell lines were sensitive to abemaciclib, with an IC50 < 1 µM. The cell lines were characterized by the loss of the 3′UTR in the *CCND1*/*CCND2*/*CCND3* genes and overexpression of *CCND2*/*CCND3* [113]. Coupled with these basic research results, CDK4/6 inhibitors have already been widely used for estrogen receptor-positive breast cancer, showing improved treatment outcomes [114,115].

It has been reported that the combination of CDK4/6 inhibitors increases the sensitivity of AML cells to cytarabine [116]. Consequently, phase 1 or 1b/2a trials of palbociclib alone or in combination with other agents for relapsed/refractory AML are ongoing.

## 5. Antibody–Drug Conjugates for AML

### Gemutuzumab Ozogamicin

The CD33 antigen is a transmembrane protein with a molecular weight of 67 kDa expressed on myeloid cells but not on mature granulocytes or hematopoietic stem cells. Gemtuzumab ozogamicin (GO), a CD33 antibody–calicheamicin conjugate, is taken up by cells after binding to CD33, and exerts its effects by cleaving double-stranded DNA in lysosomes, causing cytotoxicity and apoptosis [117]. It is believed that free calicheamicin is 1000 times more effective than doxorubicin as an antitumor agent, and many clinical trials have evaluated its efficacy for untreated and relapsed/refractory AML [118,119].

A total of 595 patients with untreated AML aged between 18 and 60 years were randomly assigned to the standard treatment group of daunomycin 60 mg/m^2^/day for 3 days and cytarabine 100 mg/m^2^/day for 7 days, or to a GO group of daunomycin 45 mg/m^2^/day for 3 days and cytarabine 100 mg/m^2^/day for 7 days with GO 6 mg/m^2^/day for 1 day (SWOG S0106 trial [120]). Patients who reached CR received cytarabine 3 g/m^2^ every 12 h on Days 1, 3, and 5. Patients who maintained CR were then re-randomized to no additional treatment or GO (GO 5 mg/m^2^ 3 times in 28 days). The CR rate between the standard treatment group and GO group was similar (70% vs. 69%), but there was a significantly higher rate of treatment-related mortality during remission-induction therapy in the GO group (1% vs. 5%). The five-year relapse-free survival and OS rates were similar between the two groups, and GO after consolidation therapy did not improve the relapse-free survival. Based on the above, it was determined that the addition of GO to standard chemotherapy for the treatment of newly diagnosed AML was not effective, and GO was temporarily withdrawn from approval by the U.S. Food and Drug Administration (FDA) in 2010.

A prospective study compared the standard treatment with GO in 1113 patients with untreated AML aged 0–71 years. The GO group received GO 3 mg/m^2^ on the first day of treatment (UKMRC AML-15 study [121]). Overall, there was no difference in CR rate, treatment-related mortality during remission induction therapy, or five-year cumulative incidence of relapse rate between the standard treatment and GO groups. However, the OS rate was significantly better in the favorable chromosomal karyotype group, at 79% in the GO group vs. 51% in the standard treatment group. In this study, a model was proposed to predict which group would show the greatest benefit in OS for GO, incorporating age, OS, and performance status as factors. OS was similar between the standard treatment and GO groups in the presence of *FLT3*-ITD and/or *NPM1* mutation-positive AML [122]. Another retrospective study suggested the efficacy of the GO combination for *KIT* exon 17 mutation-positive AML [123]. In the ALFA-0701 study [124], 280 patients with newly diagnosed AML aged 50–70 years were randomized into groups receiving standard chemotherapy or the combination of standard chemotherapy and GO 3 mg/m^2^. The primary endpoints of the 2-year EFS were estimated as 17.1% in the standard chemotherapy group and 40.8% in GO group, demonstrating the benefit of the GO combination. In the final analysis [125], which extended the observation period, the benefit of the GO combination was also observed in the 3-year EFS. Based on these results, GO admission was again approved by the U.S. FDA in 2017 for newly diagnosed and relapsed/refractory AML.

A meta-analysis reviewed 3325 patients with untreated AML who participated in several clinical trials [121,124,125]. The CR rate and mortality within 30 days of treatment were similar with and without GO. There was no difference in relapse or survival between the GO 3 mg/m^2^ and GO 6 mg/m^2^ groups. However, the GO 6 mg/m^2^ group had significantly higher mortality within 30 days of treatment [121]. Although the optimal dosing regimen for GO has not yet been established, based on the results above, we believe that 5–6 mg/m^2^ may cause severe toxicity, and that it is preferable to start dosing at 3 mg/m^2^ and administer additional doses according to the tumor burden and/or treatment response.

The NCCN guideline recommends GO for CD33-positive patients with favorable or intermediate karyotypes and as an option for post-remission therapy. In this setting, a combination with high-dose cytarabine is desirable. Based on the results of a single-center retrospective analysis, the administration of GO prior to allogeneic transplantation has been considered to be a risk for the development of sinusoidal obstruction syndrome/sinusoidal obstruction syndrome (VOD/SOS) [126]. A short period of time between GO administration and allogeneic transplantation has also been considered to be a risk factor for the development of VOD/SOS [127]. However, with the recent trend toward the use of less intense conditioning regimens and the use of reduced or fractionated doses of GO, it is now believed that GO administration prior to allogeneic transplantation should not necessarily be avoided [128]. When GO is administered prior to transplantation, it is recommended that it be started at a low dose of 3 mg/m^2^/dose and followed by supportive care such as defibrotide administration and supplemental fluid management.

## 6. Immunotherapy for AML

### 6.1. Chimeric Antigen Receptor T-Cell Therapy

T lymphocytes artificially expressing chimeric antigen receptors by gene transfer technology are called CAR-T cells. CAR-T therapy is a treatment method where CAR-T cells are cultured in vitro and infused into patients. Several clinical trials have demonstrated the efficacy of CAR-T therapy for B-cell hematological malignancies targeting CD19 antigens [20,21,22,129,130,131,132,133]. However, the clinical application of CAR-T therapy for AML lags behind that of CAR-T therapy for B-cell hematological malignancies, as the target antigens expressed on the surface of leukemia cells are often also expressed on normal bone marrow cells, and the expression of the target antigens on leukemia cells is weak.

As mentioned above, the CD123 antigen is highly expressed on leukemic cells, making it a potential surface antigen for CAR-T cells—several clinical trials are currently evaluating this [134]. Among them, two patients with AML out of six patients with four to seven previous regimens were able to undergo allogeneic transplantation with CR. One patient maintained CR, one patient had morphological leukemia-free status, and two petients had blast reduction but did not go into remission. No grade 3 cytokine release syndrome was observed, and no myelosuppression was observed [135]. With this information, we look forward to future developments.

### 6.2. Bispecific Antibody-Based Molecule

The CD123 antigen is an IL-3 receptor α-chain that is highly expressed in from 45% to 75% of patients with AML, and also on leukemia stem cells [136,137]. AML with a high expression of CD123 is considered to be at risk for primary induction failure (PIF), and is associated with a poor prognosis [138]. Flotetuzumab is a bispecific DART antibody against CD123 and CD3ε. A clinical trial was conducted on AML patients with PIF or CR duration less than 6 months. The recommended phase II dose (RP2D) is 500 ng/kg/day in a 28-day cycle [139]. The overall response rate was 30.0%, and the median OS was 10.2 months in patients who achieved CR/CRh. The most frequent adverse events were infusion-related reactions (IRR)/cytokine release syndrome (CRS), predominantly grade 1–2. Severe IRR/CRS were successfully prevented by staged dosing at week 1, pretreatment dexamethasone, rapid use of tocilizumab, and temporary dose reduction/interruption.

## 7. Future Direction

In AML treatment, the analysis of genetic abnormalities and chromosomal information is necessary for subsequent prognosis prediction and treatment decisions. In AML, in which an average of 2.76 genetic abnormalities are detected per patient at initial diagnosis [140], satisfactory results have not been obtained with single-targeted therapy, such as ABL1 inhibitors in CML. Single-cell analysis has been used to examine the clonal architecture [141], and it has been shown that the clonal heterogeneity may change dynamically, depending on whether there is already a resistant clone from the initial diagnosis or the selection pressure associated with therapeutic intervention. It is necessary to confirm whether there is already a resistant clone at initial diagnosis or whether the clonal heterogeneity dynamically changes with the selective pressure of therapeutic intervention [142].

Such analytical methods have not been widely used due to the lack of international standardization, high cost, and limited number of facilities that can perform these analyses. In addition, it is known that it takes several days to disclose the results; however, the impact of the time from diagnosis to treatment initiation on prognosis has not yet been evaluated [143,144,145,146].

Allogeneic HSCT remains to be one of the most effective treatments for long-term disease control and the eradication of leukemia stem cells. Several studies have reported the efficacy of tyrosine kinase inhibitors (TKIs) as maintenance therapy after allogeneic transplantation [147,148,149,150]. It has been reported that the use of TKIs after allogeneic HSCT enhances not only the antitumor effect but also the graft vs. leukemia effect [151]. Although we do not know the optimal timing and dose, various new targeted agents, such as TKIs and HMAs, have the potential to be used for maintenance therapy after allogeneic transplantation. At that time, biomarker studies might have been performed to identify effective patient groups, and post-transplant maintenance therapy may be individualized.

## 8. Conclusions

Until now, due to the lack of advantages of response-oriented individualized induction therapy over fixed-schedule induction therapy [152], AML has been treated as one-size-fits-all. However, in addition to stratification based on chromosomal and genetic information, individualization is now required based on patient factors such as eligibility for intensive chemotherapy and changes in clonal genetic heterogeneity during treatment.

## Figures and Tables

**Table 1:** European LeukemiaNet 2017 risk stratification by genetics.

Risk Category	ELN 2017 Risk Stratification by Genetics, NCCN Guidelines Version 3.2021
Favorable	t(8;21)(q22;q22.1); *RUNX1-RUNX1T1* inv(16)(p13.1q22) or t(16;16)(p13.1;q22); *CBFB-MYH11* Mutated *NPM1* without *FLT3-ITD* or with *FLT3-ITD* ^low^ Biallelic mutated *CEBPA*
Intermediate	Mutated *NPM1* and *FLT3-ITD* ^high^ Wild-type *NPM1* without *FLT3-ITD* or with *FLT3-ITD* ^low^(without adverse-risk genetic lesions) t(9;11)(p21.3;q23.3); *MLLT3-KMT2A* Cytogenetic abnormalities not classified as favorable or adverse
Poor/Adverse	t(6;9)(p23;q34.1); *DEK-NUP214* t(v;11q23.3); *KMT2A* rearranged t(9;22)(p34.1;q11.2); *BCR-ABL1* inv(3)(q21.3q26.2) or t(3;3)(q21.3;q26.2); *GATA2, MECOM(EVI1)* -5 or del(5q); -7; -17/abn(17p) Complex karyotype, monosomal karyotype Wild-type *NPM1* and *FLT3-ITD* ^high^ Mutated *RUNX1* Mutated *ASXL1* Mutated *TP53*

**Table 2:** Summary of approved drugs for acute myeloid leukemia.

Cytotoxic Agents		FDA Approve	Outcome	Common or Remarkable Adverse Events
CPX-351	Newly diagnosed therapy-related AML or AML with MRC	✔	median Overall Survival	myelosuppression, febrile neutropenia Pneumonia, hypoxia
			9.56(GO) vs. 5.95 months(chemo)	
Gemutuzumab ozogamicin	Newly diagnosed CD33 positive AML in combination with chemotherapy	✔	3-year Event Free Survival	myelosuppresion, veno-occlusive disease
			39.8%(GO+chemo) vs. 13.6%(chemo)	
	Relapsed/refractory CD33 positive AML	✔	median survival; 5.4 months	myelosuppresion, elevation of billirubin and hepatic transaminase
			CR/CRp; 28%	
Genetic target therapy				
FLT3 inhibitors				
Midostaurin	Newly diagnosed *FLT3-ITD* and/or *FLT3-TKD* positive AML in combination with chemotherapy	✔	median Overall Survival	myelosuppresion, febrile neutropenia iarrhea, rush/desquamation
			74.7(Mido+chemo) vs. 25.6 months(chemo)	
Gilteritinib	Relapsed/refractory *FLT3-ITD* and/or *FLT3-TKD* positive AML	✔	median Overall Survival	myelosuppresion, febrile neutropenia diarrhea, elevation of hepatic transaminase
			9.3(Gil) vs. 5.6 months(chemo)	
			CR/CRp; 34% vs. 15.3%	
Quizartinib	Relapsed/refractory *FLT3-ITD* positive AML	Only approved in Japan	median Overall Survival	myelosuppresion, febrile neutropenia, electrocardiogram QTc prolongation
			6.2(Qui) vs. 4.7 months(chemo)	
			CR/CRp; 48% vs. 27%	
IDH inhibitors				
Ivosidenib	Newly diagnosed *IDH1* mutated AML	✔	median Overall Survival; 12.6 months	diarrhea, fatigue, nausea, decreased appetite, differentiation syndrome
			CR/CRp; 42.4%	
	Relapsed/refractory *IDH1* mutated AML	✔	median Overall Survival; 8.8 months	electrocardiogram QTc prolongation, differentiation syndrome, myelosuppresion
			CR/CRi/CRp; 34.4%	
Enasidenib	Relapsed/refractory *IDH1* mutated AML	✔	median Overall Survival; 9.3 months	elevation of billirubin, differentiation syndrome, myelosuppresion, decreased appetite
			CR/CRi/CRp; 26.6%	
Non-genetic target therapy				
BCL-2 inhibitor				
Venetoclax	Newly diagnosed AML	✔	median Overall Survival	myelosuppresion, febrile neutropenia pneumonia, nausea, constipation, diarrhea
			14.7(VEN+AZA) vs. 9.6 months(AZA)	
			median Overall Survival	myelosuppresion, febrile neutropenia pneumonia, nausea, constipation, diarrhea
			7.2(VEN+LDAC) vs. 4.1 months(LDAC)	
